# Editorial: Advancing excellence in thoracic oncology: breakthroughs and impactful discoveries

**DOI:** 10.3389/fonc.2026.1950344

**Published:** 2026-08-11

**Authors:** Sharon R. Pine, Guiseppe Giaccone

**Affiliations:** 1 Division of Medical Oncology, Department of Medicine, University of Colorado Anschutz Medical Campus, Aurora, CO, United States; 2 Department of Hematology and Medical Oncology, Weill Cornell Medical College, New York, NY, United States

**Keywords:** lung cancer, biomarkers, immunotherapy, precision oncology, targeted therapy

Thoracic oncology has been transformed by discoveries that seemed impossible just a couple decades ago. Lung cancer classification has shifted from histology to molecular drivers that directly determine treatment. Immune checkpoint inhibition has moved from metastatic disease to intent-to-cure, and into the neoadjuvant setting. Sophisticated antibodies now deliver drugs or redirect immune cells, and selected patients with early-stage lung cancer can undergo less extensive surgery without sacrificing outcomes. However, the increasing number of available therapies has exposed a new challenge for the field. Future progress depends on choosing the right intervention at the right disease state, defining when the disease has changed, and ensuring that advances are equitably available across social and geographical boundaries.

Collectively, the 12 contributions to this Research Topic describe some of the most important advances, in a field that is moving away from histological classifications and uniform treatment toward an adaptive model of care. Molecular features determine treatment which is extending across disease stages and lessons are being transferred between malignancies. This Research Topic highlights that precision medicine, continued monitoring, and scientific innovation will achieve their greatest impact when they expand access, appropriately guide clinical management, and deliver durable benefit.

The success of biomarker-directed therapy is most evident in classical EGFR-mutant non-small cell lung cancer (NSCLC). Pan and Ramalingam open this collection with a review of the rapid expansion of treatment beyond the initial use of EGFR tyrosine kinase inhibitors, to including perioperative therapy (1), evolving first-line strategies in metastatic disease, and rational therapeutics following progression on osimertinib. The trajectory of EGFR-mutant disease while on treatment illustrates both the power and the limitations of precision oncology. An actionable driver can define an effective initial treatment, but in most cases it is not a curative therapeutic solution, at least in the metastatic setting.

Tumors evolve through primary on-target resistance, bypass signaling, histologic transformation, and transcriptional reprogramming, which may coexist within the same patient. The challenge has shifted from merely targeting EGFR to determining how long its control can be sustained and how the next treatment should be selected. Combinatorial strategies with chemotherapy and/or molecules targeting mechanisms of resistance (e.g. c-Met amplification) have shown improvement in survival in advanced disease, albeit at the cost of increased toxicity. These recent advances reflect the dynamic nature of the therapeutic landscape for EGFR-mutant NSCLC, which is becoming increasingly individualized, mechanism-informed, in efforts to achieve durable systemic and intracranial disease control, which is still often a major site of recurrence.

Fortman and Qin place this challenge within the broader biomarker landscape of advanced NSCLC. Their review highlights the increasing importance of comprehensive molecular testing and the range of methods that are required to conclusively predict biomarkers for targeted therapy and immunotherapy. The practical question, however, is not just whether testing should be performed, it is whether testing is sufficiently comprehensive, timely, reproducible, and accessible to influence care. The increasing number of validated targets and drugs against new targets, make it challenging to have timely inclusion of these markers in standard pathology laboratories. While some large academic institutions have developed their own in-house capabilities, most of the non-academic centers rely on an array of vendors with different platforms. Tissue scarcity, variable assay performance, long turnaround times, incomplete testing, and uncertain biomarkers of response continue to separate technical capability from clinical impact. As the number of relevant biomarkers grows, comprehensive testing should be regarded as an integral component of treatment decisions.

The therapeutic history of KRAS-mutant NSCLC teaches us a complementary lesson. Once regarded as an undruggable oncogenic target, KRAS has become a direct therapeutic dependency. Nichols and Herzberg review the developmental timeline of inhibitors of KRAS G12C, the most common form of KRAS mutation in adenocarcinoma of the lung. They review also the recent expansion of strategies directed toward other RAS alleles and regulatory states. The successful development and FDA approval of small molecule KRAS G12C inhibitors has been scientifically impressive, after decades of failed attempts (2), however, response rates are lower than those seen with typical oncogene addicted lung cancer (e.g. EGFR mutations, ALK and ROS translocation, etc.) and duration of response is shorter. The effectiveness of single-agent inhibition could be dampened by adaptive signaling, co-occurring genomic alterations, allele-specific biology, and acquired resistance. The next phase of RAS therapeutics will require more than new and more potent inhibitors. It will need combinations, larger selection of molecularly defined populations, and careful attention to whether earlier intervention can improve outcomes.

The need to interpret lung cancer response more dynamically is developed by Oner et al., who reviewed liquid biopsy for the detection, characterization, and interception of resistance across thoracic malignancies. Circulating tumor DNA and circulating tumor cells offer an ability to sample disease longitudinally, capture aspects of temporal and spatial heterogeneity, identify molecular residual disease, and potentially detect molecular progression before it becomes radiographically apparent (3). This possibility changes the purpose of biomarker analysis. Molecular profiling is no longer limited to making decisions on an initial treatment, rather, it is becoming also a tool to monitor tumor evolution. Although the use of liquid biopsies in early detection is the holy grail, significant refinements in technologies are still required before their implementation in clinical practice. Prospective trials in which biomarker-defined intervention is the central test rather than just assay performance, should help clarify the best path.

Scientific innovation is also changing the form that targeted therapy can be applied. Lee and Lovly describe antibody-based therapeutic platforms capable of delivering cytotoxic payloads, engaging immune effector cells, carrying radioisotopes, or combining more than one targeting function. Several antibody-drug conjugates (ADCs) against different targets (i.e. Her2, c-Met, TROP2) have already been approved by FDA, and a large number of ADCs against the same or novel targets are currently being developed (4). Activity of ADCs does not depend on antigen expression alone. Target density and heterogeneity, internalization, linker stability, payload properties, bystander effects, and host and tumor context all contribute to therapeutic efficacy. This complexity suggests that antibody-based agents as a single drug class or assuming that a measurable target is necessarily a sufficient biomarker need more refinement. Rational development will require assays that define the complete biology of the disease, not only the presence of its nominal antigen. Furthermore, side effects, which typically include some chemotherapy-like toxicity in addition to target specific toxicity and ADC toxicity, often require intensive premedication strategies. These side effects may hamper combinatorial strategies.

Small cell lung cancer (SCLC) offers a striking setting in which some of these ideas converge. Parmar et al. frame SCLC through lessons that can be drawn from hematologic malignancies, emphasizing lineage dependence, cellular plasticity, antigen escape, immune dysfunction, and opportunities for antigen-directed therapy. This comparison is valuable because it moves the discussion beyond the repeated addition of cytotoxic regimens to a disease that resists durable therapeutic response. Neuroendocrine and non-neuroendocrine state transitions may change both antigen expression and therapeutic vulnerability, making a fixed treatment strategy less effective. Circulating tumor DNA and epigenomic profiling provide noninvasive approaches to monitor tumor evolution, lineage state, and treatment response over time, offering potential for biomarker-guided therapeutic adaptation. The recent success with DLL3-directed therapies supports the value of lineage-associated targets, but the challenges of heterogeneity and escape still need to be addressed. This review also reports initial results with other emerging promising targeted therapies, such as ADCs against B7-H3 and SEZ6, and others (5). Our experience with hematologic malignancies suggests that sequential monitoring, rational combinations, and strategies designed to prevent or limit lineage shifting may be as important as the choice of the initial target.

The review by Leunissen et al. extends molecular profiling to rare lung neuroendocrine neoplasms, including pulmonary carcinoids and large-cell neuroendocrine carcinomas (LCNEC), are heterogeneous tumors. These tumors comprise biologically and clinically distinct entities, and small cohorts and inconsistent classification have limited the development of disease-specific treatment (6). Multi-omics studies have demonstrated that pulmonary carcinoids comprise distinct molecular subgroups (A1, A2, B, and supra-carcinoids) with unique clinical and genomic features. In clinical practice, a biomarker panel consisting of OTP, CD44, and Ki-67 could improve the prediction of disease recurrence in pulmonary carcinoids, enabling personalized follow-up strategies. Within LCNEC, two major subgroups (SCLC-like and NSCLC-like) are emerging, with targeted therapies such as DLL3 and SEZ6 showing potential for clinical application. In conclusion, the integration of subgroup-specific molecular markers into routine diagnostics is essential. Molecular characterization of these rare tumors may improve diagnostics and identify clinically relevant subgroups. To achieve meaningful clinical benefit, multi-institutional efforts such as collaborative registries, centralized pathology, shared molecular datasets, and carefully designed basket or platform studies will be necessary.

A similar need for biologically informed treatment decisions is evident in pleural mesothelioma, another uncommon thoracic tumor. van Genugten et al. review recent developments in our understanding of the biology of this tumor, its molecular hallmarks, and the shift from our longstanding reliance on platinum-based chemotherapy toward immunotherapy with dual immune-checkpoint blockade. Despite the improvement in survival obtained with dual blockade, this is primarily observed in the non-epitheliod histological subtypes, and side effects of dual immune-checkpoint blockade needs to be carefully considered (7). Emerging therapies such as new angiogenesis inhibitors, antibody-drug conjugates, bi-specific antibodies, chimeric antigen receptor T-cells, cancer vaccines, and oncolytic viruses are also discussed. Despite improved biological understanding, targeted therapies have not yet achieved routine clinical use in mesothelioma. Genomic characterization can identify recurrent alterations without yielding readily druggable targets. In diseases driven by tumor-suppressor loss, such as pleural mesothelioma, complex microenvironmental interactions, and marked heterogeneity, therapeutic development may need to focus on synthetic lethal vulnerabilities, the immune context, and combinatorial strategies rather than searching for a single dominant oncogenic target.

Pardini et al. address another rare thoracic malignancy through a systematic meta-analysis of next-generation sequencing data from thymic epithelial tumors. Their analysis supports the presence of biologically distinct molecular subgroups, including a relatively indolent GTF2I-mutant group (8), a more aggressive TP53-mutant group enriched for proliferative and receptor tyrosine kinase signaling, and a double-negative group that is characterized by extracellular-matrix, metabolic, and epigenetic features. These findings support the need to move beyond histology, however, despite a better understanding of the molecular biology of these very rare tumors, unfortunately so far there has been no significant advancement in the development of targeted therapies. In addition, retrospective datasets, differences in sequencing platforms, limited sample sizes, and the rarity of these tumors have continued to limit immediate therapeutic translation. Final determination of the value of molecular subgrouping would depend on prospective validation and if the subgroups predict prognosis or treatment response better than pathological classification.

The advances in thoracic oncology are not limited to extensive disease. Abodunrin et al. review the incorporation of immune checkpoint inhibitors into neoadjuvant, adjuvant, and perioperative treatment for resectable NSCLC. Perioperative treatment trials have revolutionized the treatment of resectable NSCLC. The addition of immune-checkpoint inhibitors to chemotherapy has in fact become standard in the neoadjuvant treatment of early NSCLC (9). Despite a relatively large number of trials in early disease setting, we still need to define which component of perioperative therapy is beneficial, in particular if postoperative treatment is necessary, especially after a complete pathological response to neoadjuvant therapy, and how molecular residual disease (ctDNA) should influence these decisions. The field lacks single biomarkers that can identify who would benefit from perioperative therapy, who can safely receive less treatment, and who is at high risk despite a major pathological response. Tumor response, circulating tumor DNA, PD-L1 expression, genomic features, radiomics, and digital pathology are all being investigated to help resolve this question. The success of perioperative immunotherapy has led to the idea that de-escalation is as important as escalation. Sustained or more intensive treatment is not always the best route, particularly when patients may already have been cured by surgery or when immune-related toxicity outweighs the benefits.

Chow et al. review the results of the CALGB 140503 (Alliance), which compared sublobar resection (wedge or segmentectomy) with lobectomy for peripheral, clinically node-negative NSCLC ≤2 cm (10). The noninferiority of sublobar resection to lobectomy showed in this trial and in the Japanese trial JCOG 0802/WJOG 4607L, has now been established sublobar resection as an acceptable – and for some patients preferable – strategy for stage IA (≤ 2 cm). The trial did not support indiscriminate reduction of surgery. Rather, tumor size and location, adequate margins, and rigorous nodal assessment were central to its conclusions. As screening identifies more lung cancers at an earlier stage, systemic therapy will continue to shift earlier, and surgical decisions will increasingly require integration with tumor biology, functional preservation, and the possibility of subsequent primary lung cancers. Interestingly, CALGB 140503 showed an unexpectedly high incidence of recurrence observed in both arms of the trial and more than 50% of these recurrences were systemic in nature, which provides compelling evidence to include these patients in neoadjuvant or adjuvant trials according to the authors.

The scientific and clinical gains highlighted in this Research Topic and beyond cannot be separated from the populations in which they are implemented. Casazza et al. trace the history of lung cancer disparities in the United States and discuss that advances in prevention, screening, diagnosis, and treatment have not been distributed equally. Rural settings, low socioeconomic status, structural barriers, and unequal access to evidence-based care have continued to influence who is screened, who receives complete molecular testing, who reaches a specialist, and who receives effective therapy in time. These are not peripheral implementation problems that begin after they are uncovered. They also affect the evidence base itself, the generalizability of trials, and the overall effectiveness of innovations in real-world routine practice. Equity should be treated as a scientific and clinical measure of success. A biomarker-dependent therapy cannot achieve its intended population if testing is unavailable to everyone. Lung cancer screening cannot reduce mortality among populations that cannot access screening. Complex perioperative regimens or systemic treatments may widen disparities if they require frequent long-distance travel. Even de-escalation strategies may be applied unevenly if high-quality staging, surgery, and pathology are not broadly available. The question is not only whether a new intervention works, but under what conditions it can work across the population.

Thoracic oncology has entered an era of extraordinary therapeutic advances. This Research Topic describes a field whose greatest opportunity now lies at connecting these advances. Excellence will be measured not only by what can be discovered or administered to patients, but also by whether the breakthroughs can be integrated into durable care that is available to the patients who need it.
